# Novel viruses in the families *Iflaviridae* and *Partitiviridae* associated with the common eastern firefly *Photinus pyralis*

**DOI:** 10.17912/micropub.biology.001385

**Published:** 2025-01-17

**Authors:** Seth A. Brewer, Meaghan J. Adler, Mckayla M. Martin, Paula Rozo-Lopez, Benjamin J. Parker

**Affiliations:** 1 Microbiology, University of Tennessee at Knoxville, Knoxville, Tennessee, United States; 2 Biology, University of North Carolina at Chapel Hill, Chapel Hill, North Carolina, United States

## Abstract

Fireflies are iconic insects that are under threat from environmental change. Knowledge of the viral diversity associated with natural firefly populations is important to our understanding of the basic biology of these insects and could be relevant to firefly conservation. We performed metatranscriptome sequencing of the Common Eastern Firefly ( *Photinus pyralis)* and assembled genomes for two new species of virus in the families Iflaviridae and Partitiviridae. We surveyed multiple individuals for these viruses using PCR, and we showed that both viruses are found at intermediate frequences in a natural population.

**Figure 1. Viruses associated with Photinus pyralis:**
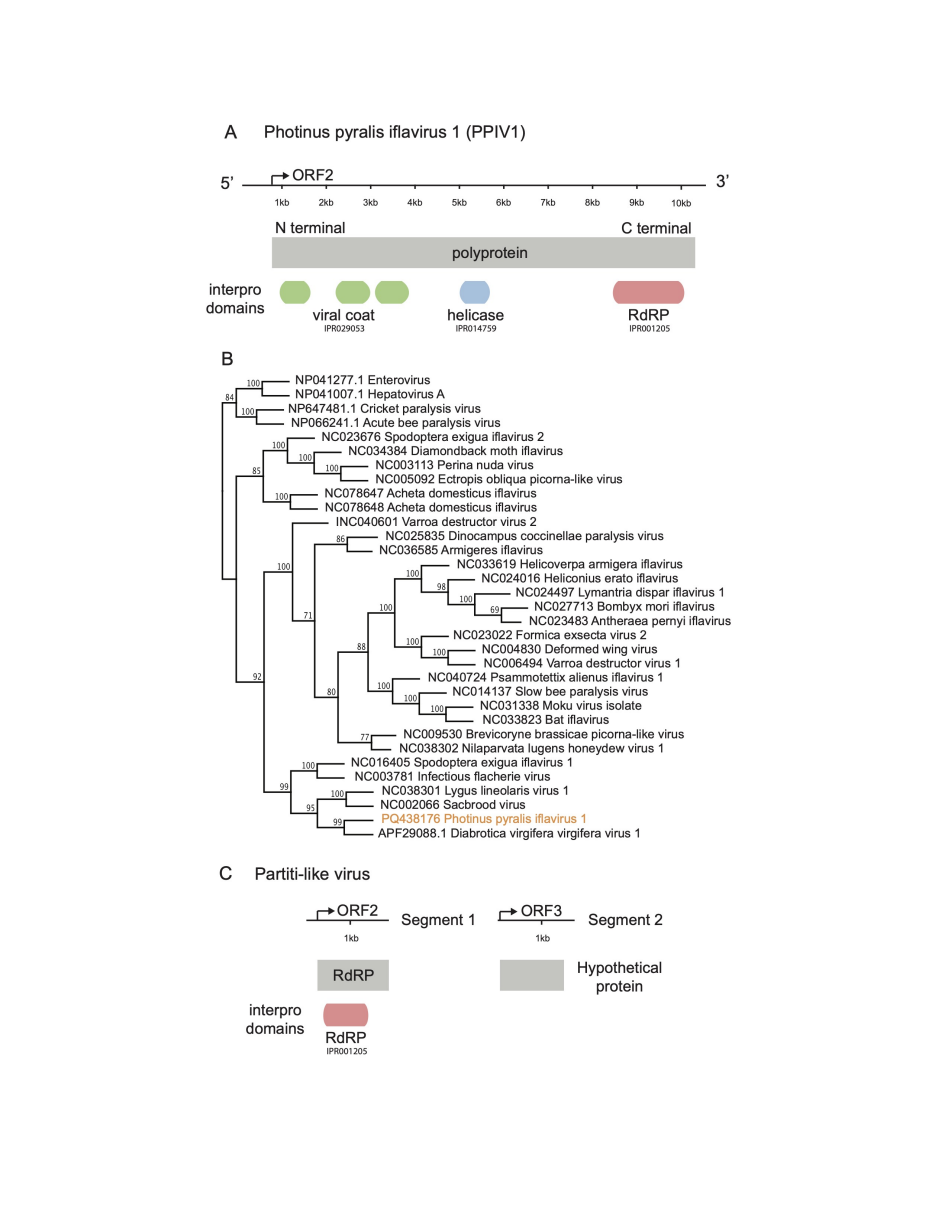
**A.** An annotated diagram of the sequence information for Photinus pyralis iflavirus 1. The top of the figure represents the 10,561bp genome with each kb indicated in the 5' to 3' direction. The location of the ORF for the polyprotein is shown, along with the extent of that animo acid sequence. The bottom of the figure shows interpro domains and accession numbers, including viral coat proteins, a helicase, and the RNA-dependent RNA polymerase. **B.** A phylogeny of insect-specific iflavirus polyprotein amino acid sequences with branch support values. PPIV1 is shown in orange. **C.** An annotated diagram of the sequence information for the partiti-like virus. The two segments are shown, along with the ORFs that encode for the RdRP and a hypothetical protein on each segment, respectively. The interpro domains and accession numbers are shown at the bottom of the figure.

## Description

In recent years, the use of next-generation sequencing has revolutionized our understanding of the diversity of viruses associated with insects. Studying insect viromes using metatranscriptome sequencing has shown that invertebrates are important reservoirs of viral genetic diversity (Li et al. 2015) , and characterizing the viromes of key insect taxa has led to insights that are relevant to pathogen spillover and insect conservation (Schoonvaere et al. 2018) . Fireflies (Coleoptera: Lampyridae) are iconic insects that have long been studied for their courtship displays and chemical ecology. Populations of fireflies are in decline, and studies have suggested that one in three North American species of fireflies is at risk of extinction (Fallon et al. 2021) . A better understanding of the potential viral pathogens associated with natural populations of fireflies is needed to aid in conservation and restoration.

In this study, we performed metatranscriptome sequencing to search for viruses in the Common Eastern Firefly ( *Photinus pyralis* ). Our data suggested the presence of two viruses and included reads with homology to “ *Diabrotica virgifera virus 1”* and reads with homology to “Partiti-like” viruses. For the first of these potential viruses (with homology to *Diabrotica virgifera virus 1* ), we assembled a 10,561 bp genome and identified a 3213 amino acid sequence translated from ORF2 in our genome ( Figure 1A ). Blastp results suggested this amino acid sequence has homology to *Iflavirus* polyproteins. Viruses in the family Iflaviridae are positive-sense single-stranded RNA viruses that are widely distributed across arthropods (Liu et al. 2015) . Iflaviruses typically have 9-11kb genomes that serve as both genome and viral mRNA; genomes are translated into a single polyprotein of around 3000 amino acids that are then processed to yield capsid proteins in the N-terminal region and non-structural proteins including a viral helicase, protease, and RNA-dependent RNA polymerase (RdRP) in the C-terminal region (Valles et al. 2017) . We used InterPro (Paysan-Lafosse et al. 2023) to search for conserved domains in the amino acid sequence, and we found multiple viral coat domains at the N-terminal end of the sequence, and a helicase and RdRP domain at the C-terminal end ( Figure 1A ).

We generated a phylogeny of the polyprotein sequence from 32 key *Iflavirus* species infecting insects ( Figure 1B ). Our phylogenetic analysis confirmed the placement of our firefly virus within other insect-associated Iflaviruses, with our sequence forming a branch with *Diabrotica virgifera virus 1* , an *Iflavirus* infecting the Western Corn Rootworm (Coleoptera: Chrysomelidae) (GenBank: KY064174.1 ). The polyprotein sequence of our virus was 28.8% similar to this closest relative, and on that basis is a new species (Valles et al. 2017) . We named this virus *Photinus pyralis iflavirus 1* (PPIV1) and we deposited the reference sequence for this new species in GenBank ( PQ438176 ). Using a PCR screen, we found that this virus infected 54% (7 out of 13) wild-collected individuals, suggesting it is found at intermediate frequencies in our local population of *P. pyralis* . We note that since PCR can produce false negatives, our estimates of field prevalence should be considered a minimum estimate and could be higher, depending on the sensitivity of the screen.

Our results also indicated the presence of reads with homology to “Hubei partiti-like virus” and “Wuhan Cricket Virus 2” in the metatranscriptome data. Partiti viruses have bi-segmented linear dsRNA genomes, with two segments that encode an RdRP and a capsid protein, respectively (Nibert et al. 2009) . We assembled an 1876 bp contig (segment 1) with a 575 amino acid protein sequence from ORF2, which had homology to Partiti-like virus RdRPs and contained an RdRP conserved domain based on an InterPro search ( Figure 1C ). We assembled a second segment with 1740 bp that contained a 506 aa sequence in ORF3 with homology to ‘hypothetical proteins' from diverse Partiti-like viruses, which we hypothesize is the capsid protein that is typical of this viral family. We deposited the reference sequences for this virus in GenBank (Segment 1: PQ438177 ; Segment 2: PQ438178 ), which we refer to as Photinus pyralis associated partiti-like virus. The RdRP amino acid sequence shares 54.3% identity to the closest relative (Hubei partiti-like virus 4; APG78224), and the amino acid sequence for the putative capsid protein is 28.3% identical to the closest viral sequence (brine shrimp partiti-like virus 1; UNI73881), which together suggest this is a new species of Partitivirus based on established criteria for species delineation in this family (Vainio et al. 2018) .

In recent years, diverse partitiviruses have been discovered in insect hosts (Cross et al. 2020) , but these viruses are also associated with fungal and plant hosts (Nibert et al. 2014) . A small number of reads in the metatranscriptome were identified as having homology to *Verticillium dahliae,* a plant-associated fungal pathogen with a broad host range. We attempted to amplify the V4 region of fungal 18S genes for amplicon sequencing, but did not get a band for any firefly samples of the expected size. Again, using a PCR screen, we found that the partiti-like virus is found at intermediate frequences in our local population (38%; 5 out of 13). Together these results suggest that this virus is associated with the firefly host rather than a fungus, but more data are needed.

This study demonstrates the utility of metatranscriptome sequencing for finding new members of established viral families, even in well-characterized insect species. Our analysis yielded the discovery of two new species of viruses associated with *P. pyralis* and provided an initial measurement of their infection frequency in a natural population. Our study did not address the transmission patterns of these new microbes or what phenotypic effects they have on hosts. Iflaviruses have been found widely across arthropods and can be pathogenic to insects but in other cases seem not to affect their hosts (Valles et al. 2017) . Partitiviruses are relatively unstudied but have been identified as heritable male-killing agents in lepidopterans (Fujita et al. 2020) . Future work is needed to determine what effects these viruses have on fireflies, their host species range, and if these are pathogens that could amplify the environmental stressors facing declining firefly populations.

## Methods

**Sample collection and sequencing** : We collected adult fireflies in Knoxville, TN, USA in June of 2023 and stored them in individual tubes at -80°C. We extracted RNA from a single individual firefly using a phenol/chloroform extraction and isopropanol precipitation, followed by cleaning and DNAse treatment with the Zymo RNA Clean & Concentrator-25 with DNase I kit. The library for next-generation sequencing was prepared using the Illumina Stranded Total RNA prep with Ribo-Zero Plus kit. We obtained 9 billion base pairs of sequencing on an Illumina NovaSeq machine with 150bp paired-end reads at Novogene, Inc. We deposited the raw reads into the NCBI Sequence Read Archive with Accession Number: PRJNA1169871 ).

**Analysis via CZ ID** : For the initial analysis of these data, we used CZ ID, an online open-access automatic pipeline for analyzing metatranscriptome data to describe viral communities using the default parameters (Kalantar et al. 2020) . We accessed this online pipeline on April 5 ^th^ 2024, and we used v.8.3 of the CZ ID Metagenomic Illumina pipeline with an NCBI index date of January 22 ^nd^ 2021. After adapter trimming and quality filtering, we removed host reads using Genome assembly Ppyr1.3 of *Photinus pyralis* (Fallon et al. 2018) . We then aligned reads to the NCBI NT and NR databases using Minimap2 v.2.21 (Li 2018) and DIAMOND v.2.0.15 (Buchfink et al. 2015) for species identification, implemented in CZ ID using default parameters. We filtered results to viruses with at least 1,000 reads per million with homology to the NR database.

**Viral genome assembly** : For assembly of both viral genomes, we used SPAdes v.3.11.1 (Prjibelski et al. 2020) implemented in CZ ID. We used the NCBI Open Reading Frame Finder (accessed April 23 ^rd^ 2024) to identify potential amino acid sequences, and blastp (accessed April 23 ^rd^ 2024) and InterPro (v.101.0) to annotate these putative proteins (Paysan-Lafosse et al. 2023) .

**Iflavirus phylogeny** : We used a MAFFT v.7.490 (Kuraku et al. 2013) alignment (FFT-NS-i_x1000, matix BLOSUM62, gap penalty 1.54) to generate a maximum–likelihood tree using RaxML (PROTGAMMAJTTF model with majority rule bootstrap with 100 reps). Families Dicistroviridae (NP647481.1 *Cricket paralysis virus* NP066241.1 *Acute bee paralysis virus* ) and Picornaviridae (NP041277.1 *Enterovirus* NP041007.1 *Hepatovirus* A) were used as outgroups.

**Iflavirus screening** : We developed primers that amplified a 500 bp region for detection of the iflavirus (F: CTCCACGGCAACAAACTTTG; R: CAAAATCACGCGAGGAACAC). We screened 13 fireflies collected from the original location using RNA extracted as above and cDNA constructed with the BioRad iScript cDNA synthesis kit. PCR reactions included OneTaq Quick-Load 2X Master Mix with Standard Buffer and primers at 10mM. PCR conditions consisted of an initial step of 95°C for 30s, 30 cycles of 95°C for 30s, 48°C for 1 minute, and 68°C for 30s, followed by a final step of 68°C for 5 minutes. We validated the PCR protocol by Sanger sequencing a representative amplicon.

**Partiti-like virus screening** : We designed primers to screen the samples for the partiti-like virus' RdRP (F: CATCGGCCGTCTTTATGTGC; R: ACAACATCAGAACTTTCCCGGA) and capsid gene (F: GGCCAGCAGACATGATTCAAC; R: TTCCTAGTCTATGGAATAAATTCCTTACTT). PCR conditions were the same as those used above for the Iflavirus. We counted a sample as virus positive if it had a band of the correct sizes for both segments. We validated the protocol via Sanger sequencing as above.
